# Preanesthetic severe postural hypotension following 5-aminolevulinic acid pretreatment in patients for photodynamic diagnosis-assisted urological surgery

**DOI:** 10.1186/s40981-019-0295-1

**Published:** 2019-11-09

**Authors:** Tohru Shiratori, Kunihisa Hotta, Masaaki Satoh, Chiaki Kiuchi, Noriyuki Ogawa, Takayuki Kamigaito

**Affiliations:** 1 Department of Anesthesiology, Ina Central Hospital, 1313-1 Koshiroukubo, Ina, Nagano 396-8555 Japan; 2 Department of Anesthesiology and Critical Care Medicine, Jichi Medical University, 3311-1 Yakushiji, Shimotsuke, Tochigi, 329-0498 Japan; 3 Department of Urology, Ina Central Hospital, 1313-1 Koshiroukubo, Ina, Nagano 396-8555 Japan

**Keywords:** 5-Aminolevulinic acid, Photodynamic diagnosis, Fluorescence, Anesthesia, Hypotension, Postural hypotension, Vasodilation, Porphyrin, Autonomic neuropathy, Bladder tumor

## Abstract

**Background:**

5-Aminolevulinic acid (5-ALA) is utilized for photodynamic diagnosis-assisted (PDD) surgery. However, it has been associated with vasodilation, hence, occasional hypotension.

**Case presentation:**

We encountered two patients who had severe postural hypotension following 5-ALA pretreatment prior to an operation. They were scheduled for urological PDD surgery, but upon standing to walk to the operation room, they felt sick because of severe hypotension. One of them underwent the surgery after recovery, but the other surgery was canceled due to a prolonged hypotension that lasted for more than a day.

**Conclusions:**

Severe postural hypotension may develop as a result of the high concentration of porphyrin precursors, which may affect the nervous system. Severe postural hypotension may be due to 5-ALA-induced autonomic dysfunction as well as vasodilative action of 5-ALA. These observations suggest that in addition to the careful monitoring of patients’ vital signs, standing should be avoided following 5-ALA pretreatment.

## Background

5-Aminolevulinic acid (5-ALA) is a precursor of protoporphyrin IX (PpIX), which has high tumor selectivity and photoactivity, leading to more accurate visualization of cancer tissues. 5-ALA is utilized for photodynamic diagnosis-assisted (PDD) glioma or bladder tumor surgery, and it is orally administered 3 h before the surgery [1–3]. Although hypotension is among the adverse events associated with 5-ALA, it has not been well recognized as serious [1–3]. This may partly explain why the characteristics, clinical course, and mechanism of 5-ALA-induced hypotension have not been fully elucidated. A recent case report described the anesthetic course of 5-ALA-induced severe hypotension that occurred immediately after the induction of general anesthesia [4]. Here, we report two cases of severe postural hypotension following 5-ALA pretreatment for transurethral resection of bladder tumor (TURBT).

## Case presentation

### Case 1

A 75-year-old man (height 165 cm, weight 63 kg) was scheduled to undergo his third TURBT. For prostatic hyperplasia, he was prescribed 5α-reductase inhibitor. Preoperative examinations were within the normal ranges. Systolic blood pressure (S-BP) was about 140 mmHg at home. On the patient’s request, general anesthesia was planned. On the day of the operation, the BP was 139/82 mmHg. A nitroglycerin transdermal patch (nitroglycerin 25 mg) was used at the start of preoperative crystalloid infusion (100 mL/hr) as a routine medication against perioperative myocardial ischemia. For the first time in his life, the patient took a 5-ALA (1.5 g) solution 3 h before TURBT but felt sick afterwards. About 2 h later, the patient walked to the lavatory, but upon returning, he could not sit by himself and had a severe staggered feeling and nausea. Although his S-BP was 42 mmHg, he was fully conscious. The patient had cold sweats and a pulse rate (PR) of 70–80 bpm, but other skin symptoms were absent. The blood glucose level was 146 mg/dL. Placing the patient in the Trendelenburg position with fluid resuscitation brought recovery from the shock. Before entering the operating room, the BP and PR were 96/55 mmHg and 67 bpm, respectively. General anesthesia was induced with atropine (0.5 mg), propofol (80 mg), and rocuronium (30 mg). Anesthesia was maintained with sevoflurane (1.0–1.5%) and remifentanil (100–300 μg/h). Although ephedrine (total dose 15 mg) and phenylephrine (total dose 0.2 mg) were necessary to maintain S-BP above 80 mmHg, abnormal hypotension did not develop during the PDD surgery. The operation was completed without any incident, and the postoperative course was uneventful. However, the pathology report suggested the need for a future PDD surgery. With the nitroglycerin patch attached, the hypotension seemed non-ischemic. Although the nitroglycerin patch could have affected the preoperative BP, it was difficult to conclude that the patch caused the severe hypotension.

### Case 2

A 68-year-old man (170 cm, 70 kg) was planned for his first elective PDD TURBT under spinal anesthesia. The patient was medicated with losartan (25 mg/day) for hypertension and tamsulosin (0.2 mg/day) for dysuria. Preoperative examinations were within the normal ranges. On the day of the operation, the BP and PR were 126/81 mmHg and 71 bpm in the early morning, respectively. Daily medications were orally administered. Subsequently, crystalloid infusion was started. About 3 h before the PDD TURBT, the patient took a 5-ALA (1.5 g) solution. Thirty minutes before entering the operation room, the BP and PR were 96/57 mmHg and 80 bpm, respectively. When the patient stood up and headed to the operation room, he felt sick. The S-BP was 42 mmHg at that time. After 20 min, the vital signs were 76/44 mmHg and 68 bpm, respectively. He complained of dizziness with cold sweat and no rash. Fluid resuscitation with the Trendelenburg position was initiated. The operation was canceled due to sustained hypotension, and continuous dopamine infusion (0.3% solution; 3 mL/hr) was started. About 16 h after the 5-ALA pretreatment, the vital signs were 67/32 mmHg and 78 bpm, respectively. Continuous dopamine infusion was necessary until the next day. No reason was found for the hypotension except the 5-ALA pretreatment.

## Discussion

The clinical courses of the two patients suggest some important findings following 5-ALA pretreatment: first, severe postural hypotension followed 5-ALA pretreatment; second, severe postural hypotension occurred about 3 h after 5-ALA pretreatment; and third, the hypotension was severe in the abrupt onset and duration.

The clinical situations of the two patients may indicate a causal relationship between severe postural hypotension and exposure to 5-ALA, this being the common clinical factor between them. The possible causes of the postural hypotension observed in the two patients could be strong vasodilation and autonomic disorder. 5-ALA may induce vasodilative action [5]; however, it is debatable whether severe hypotension is ascribed only to vasodilative action because 5-ALA does not always induce severe BP decrease and its dose-response relationship is unclear. According to previous researches, there have been some concerns about the relationship between 5-ALA-induced hypotension and antihypertensive therapy [6, 7]. Although preanesthetic antihypertensive may be a factor spurring hypotension, it is uncertain whether the severe postural hypotension was only due to 5-ALA pretreatment in combination with the antihypertensive because 5-ALA-pretreated patients medicated together with antihypertensive have not always experienced preanesthetic severe hypotension in clinical practice. Previous trials contain no information on how patients moved to the operation room. In neurosurgical trials, patients with high-grade glioma are considered to have been laid on the bed before an operation. Postural hypotension may not have developed in neurosurgical trials because such patients hardly have the need to walk to the operation room and are usually transferred on the bed. While we have hypothesized that autonomic reflex dysfunction or strong vasodilation beyond autonomic reflex may be responsible for the severe hypotension observed in our patients, the true mechanism is not known.

The onset time of severe hypotension in our cases was approximately 3 h after 5-ALA pretreatment, similar to a recent report [4] and seems to coincide with an increase in plasma PpIX concentration. PpIX concentration slopes upward an hour after 5-ALA pretreatment, and the 3-h concentration reaches about 3 times as high in an hour [5], indicating that substantial exogenous 5-ALA yields high concentrations of porphyrin precursors, similar to porphyria in terms of high porphyrins. While it was concluded that 5-ALA does not induce porphyria [8, 9], there is a report presenting a patient with 5-ALA-induced acute neuropathy similar to porphyria who experienced recurrent postural hypotension [10]. Postural hypotension is among the signs of porphyria autonomic neuropathy, and ample 5-ALA is supposed to have direct neurotoxicity [11–13]. Considering this fact, it is possible that autonomic dysfunction develops around 3 h after 5-ALA pretreatment when the concentration of porphyrin precursors becomes extremely high. Given that substantial exogenous 5-ALA can neurogenically affect BP regulation, consultation from a neurologist may be needed.

The abrupt onset of profound hypotension and duration of BP decrease was serious; therefore, anaphylactic shock should be differentiated. 5-ALA can induce hypersensitivity. Two previous clinical studies have each listed a case with 5-ALA-related rash [1] and urticaria [3], and anaphylactic shock occurred in a patient with intravesical 5-ALA-derivative [14]. Thorough inspections for hypersensitivity may be required in severe cases [4]. According to a recent report, 5-ALA-induced hypotension may possibly be vasoactive-refractory [4]. While severe hypotension improved in the first case, the second case suffered prolonged hypotension that continued by the next day despite continuous dopamine infusion. Although it is not fully elucidated what classes of antihypertensive drugs significantly contribute to 5-ALA-induced hypotension, preoperative administration of angiotensin-receptor-blocker is known to be associated with an increased incidence of intraoperative hypotension [15, 16]. Losartan was administered in the second case, taking into account its advantages and disadvantages, but it may have been attributed to the severity and prolonged duration of 5-ALA-induced hypotension. Given that the effects of 5-ALA are strong and long-lasting, adequate vasoactives and careful monitoring over a day will be necessary when dealing with 5-ALA-induced hypotension.

When treating patients with 5-ALA pretreatment, there are some important insights elicited from the present cases. Standing should be avoided, and it is beneficial not to take vasodilatives preoperatively together with 5-ALA. Checking for vital signs is needed around 3 h after 5-ALA pretreatment. Preparation for prolonged hypotension may be required. As for 5-ALA indication, it is necessary to elucidate whether a history of 5-ALA-induced hypotension is a risk factor for the next 5-ALA pretreatment, because a previous study described recurrent symptomatic hypotension in patients with similar hypotensive histories [17]. However, the description lacks sufficient clinical details to draw generalizations.

In conclusion, we admitted two patients with severe postural hypotension following 5-ALA pretreatment. One patient managed to undergo PDD TURBT with general anesthesia after recovering from the hypotension, but the other could not undergo the procedure due to prolonged hypotension. Since 5-ALA may affect the nervous system, the severe hypotension observed in our patients may be attributable to the effects of 5-ALA-induced autonomic dysfunction. As such, careful monitoring of the vital signs during perioperative period is required after 5-ALA pretreatment and patients so treated should avoid standing. The relationship between 5-ALA and porphyrin metabolism may be investigated to unravel possible mechanisms for severe hypotension.

## Data Availability

Not applicable.

## Abbreviations

5-ALA: 5-Aminolevulinic acid
PDD: Photodynamic diagnosis-assisted
PpIX: Protoporphyrin IX
PR: Pulse rate
S-BP: Systolic blood pressure
TURBT: Transurethral resection of bladder tumor
